# Primary cerebral pheohyphomycosis in an immunocompetent adult: A case report and review of the literature

**DOI:** 10.1016/j.idcr.2026.e02808

**Published:** 2026-10-22

**Authors:** Hani Hamad, Mohammed Abu Khattab, Husam Eldin Salah, Ali Omrani, Sreethish Sasi, Muna Al Maslamani

**Affiliations:** a Infectious disease, Department of Internal Medicine, Hamad Medical Corporation, Doha, Qatar; b Division of Microbiology, Department of Laboratory Medicine & Pathology, Hamad Medical Corporation, Doha, Qatar

**Keywords:** Pheohyphomycosis, *Cladophialophora*, Fungal brain abscess, Fungal infection

## Abstract

Cerebral pheohyphomycosis is a rare, life-threatening central nervous system infection caused by dematiaceous fungi. It predominantly affects immunocompromised individuals; however, cases in immunocompetent hosts have been increasingly reported, particularly in association with environmental exposure. The diagnosis is challenging due to nonspecific clinical manifestations and radiological features that often mimic neoplastic or bacterial lesions. We report the case of a previously healthy 45-year-old Kenyan male who presented with chronic headache and progressive neurological deficits. Neuroimaging revealed multiple intracranial lesions with significant mass effect. Stereotactic biopsy and microbiological analysis identified *Cladophialophora* species as the causative organism. The patient was managed with surgical drainage and prolonged antifungal therapy using liposomal amphotericin B and voriconazole, resulting in significant clinical improvement. This case highlights the importance of considering fungal infections in patients with atypical brain lesions, even in the absence of overt immunosuppression. Early recognition combined medical–surgical management, and prolonged antifungal therapy are critical for improving outcomes.

## Introduction

Pheohyphomycosis refers to a heterogeneous group of infections caused by dematiaceous fungi characterized by melanin-containing cell walls, which confer resistance to host immune responses and antifungal agents. These organisms are widely distributed in soil, decaying vegetation, and organic debris, particularly in tropical and subtropical regions [1], [2].

Central nervous system (CNS) involvement, termed cerebral pheohyphomycosis, represents the most severe manifestation of these infections and is associated with high morbidity and mortality. The most frequently implicated organism is *Cladophialophora bantiana*, a neurotropic fungus with a predilection for brain tissue [3], [4].

Cerebral pheohyphomycosis is classically described in immunocompromised patients, such as those with HIV infection, malignancy, organ transplantation, or prolonged corticosteroid use. However, up to 50% of reported cases occur in immunocompetent individuals [5], [6]. Environmental exposure and traumatic inoculation are believed to play key roles in such cases.

Due to its rarity and nonspecific presentation, cerebral pheohyphomycosis is frequently misdiagnosed as a neoplasm or bacterial abscess, leading to delays in treatment. We report a case of primary cerebral pheohyphomycosis in an immunocompetent adult with occupational and environmental exposure, highlighting the diagnostic challenges and therapeutic considerations, **highlighting the importance of exposure history and early tissue diagnosis in patients with atypical intracerebral lesions**.

## Case presentation

A 45-year-old Kenyan male presented to the emergency department with a six-month history of progressive right-sided headache. Initially, the headache was mild to moderate, achy in nature, intermittent, and responded well to simple analgesics. However, the headache worsened significantly over few days prior to presentation, prompting him to seek medical care. Along with the headache, he reported recent onset of left-sided weakness affecting both his upper and lower limbs, which he described as mild but gradually progressive.

The patient’s background included no significant medical history, and he reported being generally healthy prior to the onset of his symptoms. He had no history of trauma, fever, nausea, vomiting, weight loss, or positional headache. He denied recent infections or contact with ill individuals. His social history was notable for being a cab driver in Qatar, though he regularly traveled to Kenya, where he spent time working on a farm handling cattle and sheep. His farming activities included cutting grass, which placed him in frequent contact with soil and decaying vegetation, known reservoirs for dematiaceous fungi.

On examination, the patient was afebrile and vitally stable. He was alert and oriented, with a Glasgow Coma Scale (GCS) score of 15/15. Neurological assessment revealed mild left-sided hemiparesis, though strength was preserved at 5/5 in all limbs. There were no sensory deficits, and cranial nerve function was intact. Cardiovascular, respiratory, and abdominal examinations were unremarkable.

A computed tomography (CT) scan of the head was performed. Imaging revealed a large intra-axial mass lesion in the right frontotemporal region, predominantly centered in the basal ganglia. The lesion had peripheral hyperdense rims with central necrosis and measured approximately 3 × 2.7 cm. Significant surrounding vasogenic edema was noted, resulting in a 1.2 cm midline shift to the contralateral side (Fig. 1).

**Fig. 1:**
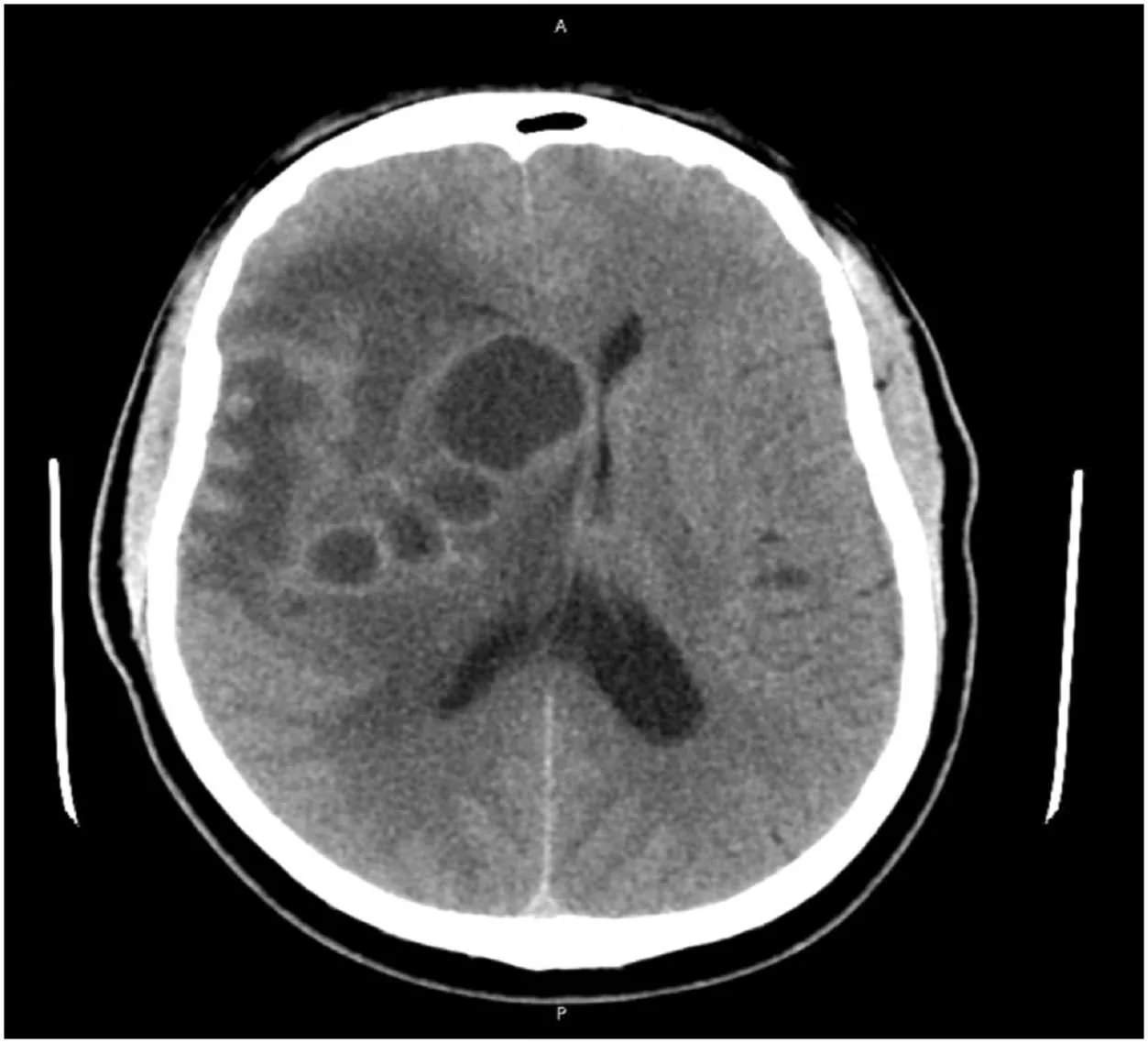
Non-contrast axial computed tomography (CT) scan of the brain demonstrating multiple intra-axial hypodense lesions in the right frontotemporal and basal ganglia regions with peripheral hyperdense rims and central necrosis, associated with extensive surrounding vasogenic edema and significant mass effect, resulting in compression of the right lateral ventricle and contralateral midline shift.

The initial working differential diagnosis included high-grade glioma or cerebral abscess. A magnetic resonance imaging (MRI) was recommended for further evaluation. Given the possibility of a cerebral abscess, empiric antibacterial therapy with intravenous ceftriaxone and metronidazole was initiated while awaiting definitive microbiological diagnosis.

MRI of the brain confirmed the presence of multiple, well-defined conglomerate lesions in the right frontal subcortical and periventricular regions, extending to the basal ganglia. These lesions exhibited fluid-like signal intensity with strong diffusion restriction and a peripheral hemosiderin rim. The largest lesion measured approximately 31 × 29 x 38 mm. Additional findings included significant vasogenic edema, effacement of the right lateral ventricle, and a 10 mm midline shift (Fig. 2). MR spectroscopy showed an elevated choline peak with increased choline-to-N-acetylaspartate (CHO/NAA) ratio and a prominent lactate peak, findings suggestive of an infectious process.

**Fig. 2:**
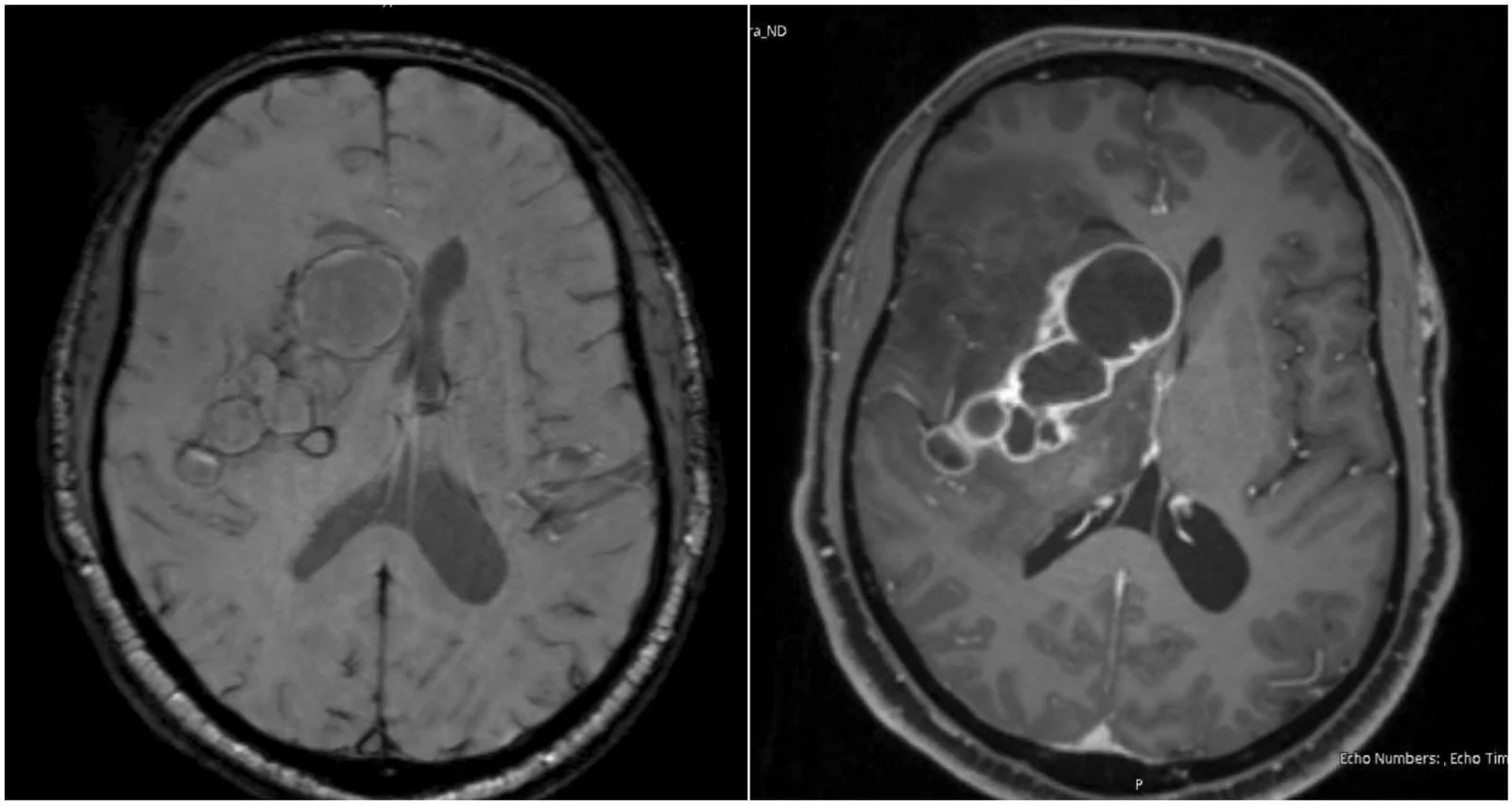
Magnetic resonance imaging (MRI) of the brain demonstrating multiple conglomerate ring-enhancing lesions in the right frontal subcortical and periventricular regions extending to the basal ganglia. Axial T2-weighted and post-contrast T1-weighted images show centrally necrotic lesions with peripheral enhancement, surrounding vasogenic edema, ventricular compression, and associated mass effect, consistent with intracerebral abscess formation.

A stereotactic biopsy of the lesion was performed to establish a definitive diagnosis. A right frontal burr hole was also created to relieve intracranial pressure and allow for abscess drainage. Intraoperatively, thick brownish purulent material was aspirated from the lesion (Fig. 3) and sent for microbiological, histopathological, and molecular analysis.

**Fig. 3:**
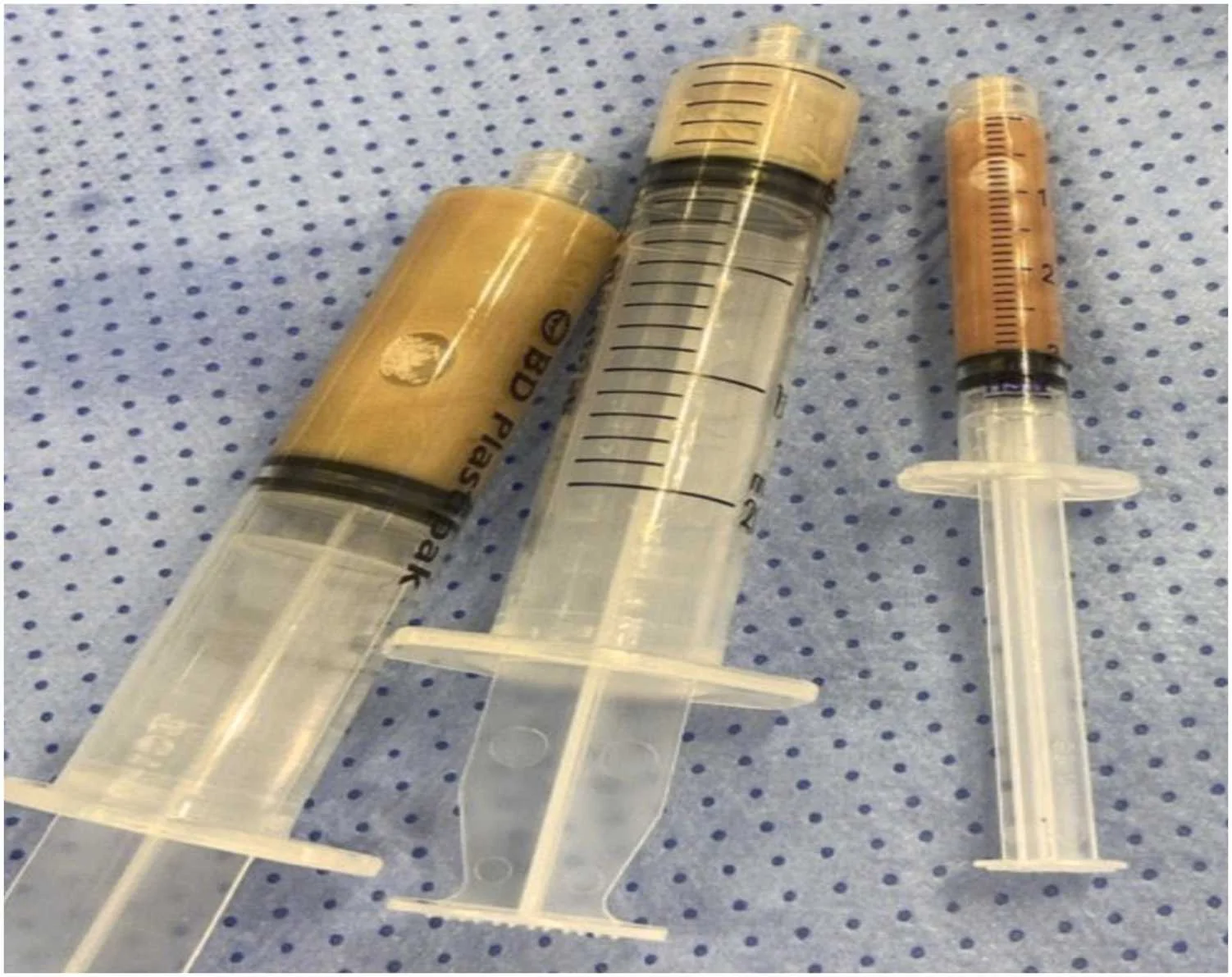
Intraoperative photograph demonstrating purulent material aspirated during stereotactic drainage of the intracerebral lesion.

Direct microscopic examination demonstrated septate, pigmented branching hyphae consistent with dematiaceous fungal infection (Fig. 4). Subsequent fungal culture identified the presence of *Cladophialophora* species based on colony and microscopic morphology, a member of the dematiaceous fungi group and a known causative agent of cerebral pheohyphomycosis. Molecular identification and antifungal susceptibility testing were not performed.

**Fig. 4:**
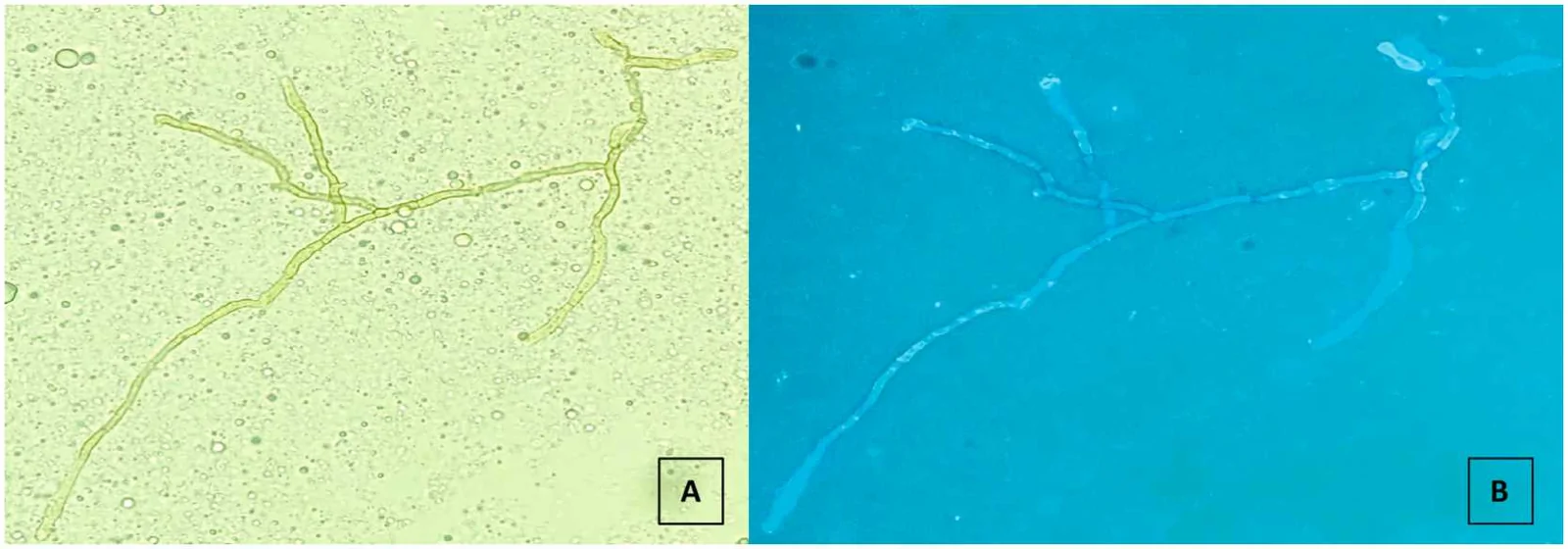
Microscopic examination of aspirated intracerebral material. A- Brown pigmented hyphae indicating dematiaceous fungi; B- Fluorescent stain demonstrating septate branching hyphae consistent with dematiaceous fungi.

Blood tests, including HIV and hepatitis screening, returned negative results, and there was no known history of an immunocompromising condition or immunosuppressive therapy. However, his hemoglobin A1c was elevated at 6.8, suggesting new-onset diabetes.

The patient was diagnosed with primary cerebral pheohyphomycosis caused by *Cladophialophora* species. This diagnosis was supported by clinical, radiological, and microbiological findings, including the identification of dematiaceous fungal elements in the CNS.

Following the confirmation of fungal infection, empirical antifungal therapy with liposomal amphotericin B (500 mg [approximately 5.7 mg/kg] every 24 h) was initiated. Intravenous Voriconazole, a preferred agent due to its CNS penetration, was added upon identification of the fungal species. Voriconazole was administered at 400 mg every 12 h for approximately 10 days, followed by 300 mg every 12 h for approximately five weeks before transition to oral therapy at 300 mg twice daily. Liposomal amphotericin B was continued for approximately seven weeks. Renal function, serum electrolytes, and liver enzymes were monitored serially throughout antifungal therapy. The patient developed acute kidney injury with hypokalemia and hypomagnesemia, attributed to liposomal amphotericin B, which were managed with intravenous fluids and electrolyte replacement. Transient elevation of liver transaminases without hyperbilirubinemia was also observed. Voriconazole therapeutic drug monitoring was not performed.

The patient demonstrated gradual clinical improvement during hospitalization, with decreased headache intensity and partial recovery of left-sided weakness. A follow-up magnetic resonance imaging (MRI) study performed one month after initiation of antifungal therapy showed mild regression of the largest lesion, with reduction of midline shift from 10 mm to 7 mm. Ventricular effacement improved; however, residual vasogenic edema and areas of restricted diffusion persisted.

The patient subsequently returned to his home country and reported continued symptomatic improvement prior to departure. He was maintained on combined antifungal therapy, with a planned treatment duration of at least six months to reduce the risk of recurrence. Ongoing follow-up with periodic clinical and radiological assessment was recommended to monitor treatment response and potential adverse effects.

At the last available follow-up, the patient remained neurologically stable, with significant reduction in headache severity and near-complete resolution of left-sided weakness.

## Discussion

Here, we report a case of primary cerebral pheohyphomycosis in an immunocompetent adult. This case highlights the significant diagnostic and therapeutic challenges associated with this rare entity. The prolonged and nonspecific clinical presentation, combined with radiological features that closely mimicked intracranial neoplasms and bacterial abscesses, contributed to diagnostic uncertainty and delayed definitive management.

Cerebral pheohyphomycosis is a rare but highly aggressive central nervous system infection caused by dematiaceous fungi, with a particularly severe course in immunocompetent individuals [1], [3], [4]. Among the causative organisms, *Cladophialophora bantiana* is the most frequently implicated species and demonstrates marked neurotropism [3], [7]. This organism possesses melanin within its cell wall, which enhances resistance to oxidative stress and host immune responses, thereby facilitating survival and invasion within neural tissue [1], [8]. Consequently, infections caused by this species are associated with high morbidity and mortality, even in previously healthy hosts [4], [5].

Although cerebral pheohyphomycosis is traditionally considered an opportunistic infection, a substantial proportion of reported cases occur in immunocompetent individuals [4], [5]. Environmental exposure appears to play a critical role in disease acquisition, particularly in tropical and subtropical regions where dematiaceous fungi are abundant in soil, decaying vegetation, and agricultural environments [1], [2]. In the present case, the patient’s frequent travel to rural Kenya and prolonged occupational exposure to farm soil, vegetation, and organic material likely contributed to fungal inoculation and subsequent central nervous system infection. This case highlights the diagnostic value of detailed environmental exposure history and early tissue diagnosis in an apparently immunocompetent patient with atypical intracerebral lesions.

Although the patient had no recognized major immunocompromising condition, an HbA1c of 6.8% indicated previously unrecognized diabetes mellitus, representing a potential host risk factor despite the relatively mild degree of glycemic elevation. The pathogenesis of cerebral pheohyphomycosis remains incompletely understood. Proposed mechanisms include inhalation of fungal spores with subsequent hematogenous dissemination, direct traumatic inoculation, and contiguous spread from paranasal sinuses [3], [6]. Once established within the brain, the organism exhibits a propensity for abscess formation, hemorrhage, and progressive local invasion, leading to significant mass effect and neurological compromise [3], [9].

Diagnosis of cerebral pheohyphomycosis is challenging due to its nonspecific clinical presentation [3], [9]. Patients commonly present with headache, seizures, focal neurological deficits, or altered mental status, which may evolve insidiously over several months [5], [9]. These manifestations closely resemble those of intracranial neoplasms, tuberculomas, or pyogenic abscesses, frequently resulting in delayed diagnosis [9], [10]. Neuroimaging findings, including ring-enhancing lesions, surrounding edema, and hemorrhagic components, are suggestive but lack specificity [10]. Therefore, definitive diagnosis relies on histopathological examination and microbiological identification of fungal elements, supported by culture and molecular techniques [3], [11].

Early tissue diagnosis is essential, as delayed initiation of appropriate antifungal therapy is a major determinant of poor outcome [4], [6]. In this case, stereotactic biopsy and drainage facilitated both diagnostic confirmation and reduction of intracranial pressure, thereby improving therapeutic response. Histopathological demonstration of septate pigmented hyphae and subsequent molecular identification of *Cladophialophora* species established the diagnosis and guided targeted therapy [7], [11].

Currently, there are no standardized treatment guidelines for cerebral pheohyphomycosis due to the rarity of the condition and the absence of randomized clinical trials [6], [12]. Management is based primarily on case series and expert opinion [4], [12]. Most authors advocate for a combined medical–surgical approach whenever feasible [3], [4], [12]. Surgical excision or drainage reduces fungal burden, alleviates mass effect, and enhances antifungal penetration [12]. Prolonged antifungal therapy remains the cornerstone of treatment [6], [13].

Voriconazole is considered a first-line agent owing to its favorable pharmacokinetic profile and excellent central nervous system penetration [6], [12]. Liposomal amphotericin B is frequently used in severe cases or during initial induction therapy [6], [13]. Combination therapy is commonly employed, although optimal regimens and treatment durations remain undefined [12], [13]. In reported cases, treatment courses often extend for six months or longer and require close monitoring for toxicity and disease recurrence [4], [12].

Despite advances in diagnostic techniques and antifungal therapy, cerebral pheohyphomycosis continues to carry a guarded prognosis, with reported mortality rates exceeding 50% in some series [4], [5]. Favorable outcomes are more likely in patients who receive early diagnosis, aggressive surgical management, and prolonged antifungal treatment [4], [12]. The relatively positive clinical course observed in this patient highlights the importance of timely intervention and multidisciplinary management.

## Conclusion

Primary cerebral pheohyphomycosis remains a rare and life-threatening condition, especially in immunocompetent individuals. In patients presenting with atypical brain abscesses, particularly with relevant environmental exposure history, clinicians should consider fungal etiologies. Early diagnosis, aggressive antifungal treatment, and surgical management are essential to improve outcomes and reduce mortality associated with this serious infection.

## Originality & exclusivity

This manuscript is original, has not been published previously, and is not under consideration elsewhere.

## Author statement

HH, MA, AO and SS were responsible for patient management and data collection. HH wrote the original draft. HES performed laboratory analysis and diagnostic confirmation. MAM supervised the case report and provided critical intellectual input. All authors contributed to manuscript drafting, revision, and approved the final version.

## Authorship & approval

− All authors listed have made substantial contributions to the work.
− All authors have read and approved the final version of the manuscript and agree to its submission.
− The order of authorship has been approved by all authors.

## CRediT authorship contribution statement

**Hani Hamad:** Writing – original draft, Investigation, Conceptualization. **Mohammed Abu Khattab:** Investigation. **Husam Eldin Salah:** Investigation. **Ali Omrani:** Supervision, Investigation. **Sreethish Sasi:** Investigation, Conceptualization. **Muna Al Maslamani:** Supervision.

## Ethics approval and consent to participate

Institutional ethics approval was not required for this case report and a waiver was obtained.

## Consent for publication

Written informed consent for publication of clinical details and anonymised images was obtained from the patient. Copy of the consent form are available to the Editor upon request.

## Funding

This publication will be supported by the Qatar National Library Open Access Fund.

## Declaration of Competing Interest

The authors declare no conflicts of interest.
